# Gangliosides and cholesterol, two major components of the membrane lipid rafts, as new regulatory partners for stress granules assembly

**DOI:** 10.1016/j.cstres.2025.100093

**Published:** 2025-07-19

**Authors:** Anaïs Aulas, Coralie Di Scala

**Affiliations:** Neuroscience Center, HiLIFE, University of Helsinki, Helsinki, Finland

**Keywords:** Lipid rafts, Cholesterol, Gangliosides, Stress granules, Stress, Translation

## Abstract

Stress granules are cytoplasmic inclusions with cyto-protective functions assembling in response to stress. They are now accepted to be part of the pathological mechanism in several diseases, from cancer to neurodegenerative disorders. However, the field is still struggling to find common regulators of their assembly and function. In this study, we describe a mechanism involving lipid rafts (gangliosides and cholesterol), in the regulation of stress granules formation. This study reports that membrane lipid composition is able to regulate the formation of stress granules potentially unraveling several disease mechanisms.

## Introduction

Along all cytoplasmic inclusions stress granules are very specific.^1^ They are not delimited by any membrane, handle a liquid-liquid phase separation driven by messenger ribo nucleic acids (mRNAs) and proteins and assemble very quickly (minutes) in response to stress exposition, and resolve upon stress removal. Their protein composition is unique, with G3BP1 and G3BP2 being the center of the protein network necessary for their assembly.^2^, ^3^, ^4^, ^5^ They help the cells to overcome from stress exposure.^2^, ^6^, ^7^, ^8^, ^9^, ^10^ Indeed, stress exposure induces a global translation repression and directs untranslated mRNA into stress granules.^11^, ^12^ This 1) protect them from stress induced degradation^6^, ^13^ and 2) give the translation priority to stress responsive proteins such as chaperones.^11^, ^14^ This action saves energy for the cells^15^ and allows the efficient translation restart as soon as stress is removed.^6^ On top, stress granules assembly protects cells from cell death by inhibiting the action of pro-death protein *via* their recruitment into the structure.^8^, ^9^, ^10^, ^16^ These properties prompt researches to investigate the link between stress granules and human diseases and how stress granules are now related to several human disorders from neurodegenerative diseases^3^, ^17^ to cancer.^16^, ^18^, ^19^

The stress granules regulation in diseases is still under intensive investigation and is directing the research to unexplored diseases-induced dysregulations. Among them, the contribution of lipid rafts in those processes have been overlooked. This is a tremendous gap in the field since lipids field have unraveled dysregulation in virtually all human pathologies. More specifically, profound lipid alterations have been observed in cancer where specific gangliosides are upregulated at the cancer cell membrane,^20^ such as GD2 in breast cancer^21^ or cholesterol increase correlates with resistance to cancer drugs.^22^, ^23^, ^24^ Several neurodegenerative diseases, like Alzheimer’s and Parkinson’s diseases, are associated with cholesterol metabolism and distribution dysregulation^25^, ^26^ that may lead to a slight increase of cholesterol level in the brain. These pathologies also show altered amounts of gangliosides such as an accumulation^27^, ^28^ of GM3 in Parkinson’s disease or increased GM1 levels in Alzheimer’s disease. Additionally, cholesterol and gangliosides regulate cell signaling in neuronal cells^29^ and cancer cells.^30^ This proves that membranes lipids composition can interfere with cell signaling. In this study we investigate the potential effect of cholesterol and gangliosides perturbing drugs in the cell ability to assemble stress granules.

## Results

**Fig. 1:**
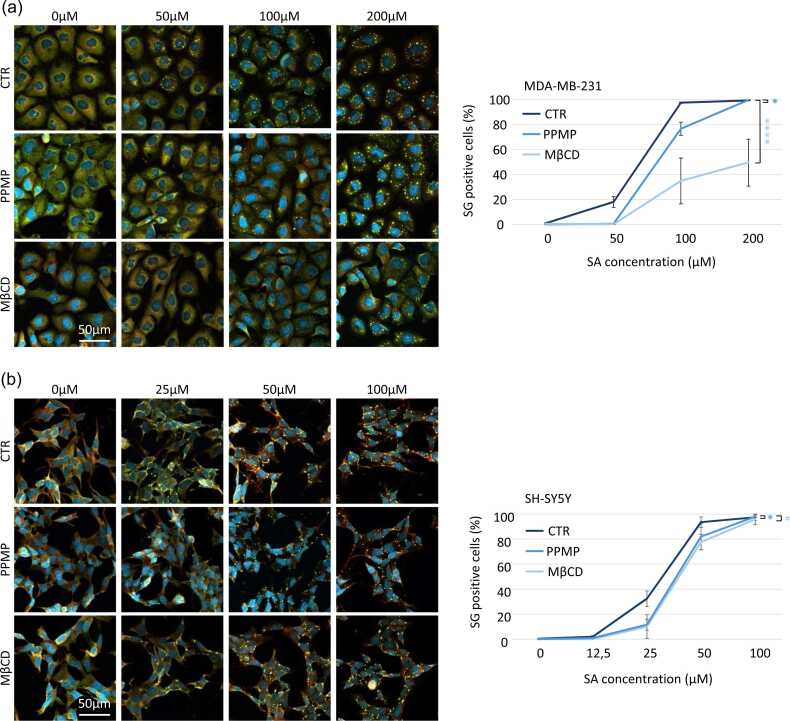
**Cells with lack of gangliosides and cholesterol at the membrane required higher dose of sodium arsenite (SA) to induce the assembly of stress granules.** (a) MDA-MB-231 cells are treated with PPMP (5 μM, 48 h) or MβCD (5 mM, 24 h) before stress granule experimentation. On the day of the experimentation, cells were treated 1 h with SA at the indicated concentration and collected. (b) SH-SY5Y cells are treated with PPMP (10 μM, 48 h) or MβCD (1 mM, 24 h) before stress granule experimentation. The day of the experimentation cells were treated 1 h with SA at the indicated concentration and collected. (a) and (b) After collection cells are then fixed and stained with G3BP1 (green), Caprin-1 (red) for stress granule labeling and DAPI (bleu) for nuclei visualization before to by imaged using confocal microscopy. Left: representative pictures. Right: quantifications. N ≤ 3, *P < 0.05, ****P < 0.0001. Abbreviation used: MβCD, methyl-β-cyclodextrin.

Stress granules are assembling in virtually all kind of cell lines from bone to white blood cells.^3^ In this study we want to investigate if stress granules regulation pathway could imply the same mechanism across tissues. We chose to investigate the potential regulation of stress granules by cholesterol and gangliosides using two cell lines: MDA-MB-231 (breast cancer cell line) and SH-SY5Y (neuroblastoma cell line) to have a proof of concept in different tissues. We subject both cell lines to an increased concentration of sodium arsenite (SA), a well-known oxidative stress inducing stress granules,^3^ to setup a baseline of sensitivity. To follow stress granules in a robust way, we follow G3BP1 and Caprin-1, two specific stress granules markers that colocalize in cytoplasmic foci^31^, ^32^ (Figure 1(a) and (b)). Each cell line has its own sensitivity, since SH-SY5Y cells start assembling stress granules at a lower SA concentration (25 μM) than MDA-MB-231 cells (50 μM) (Figure 1(a) and (b)). Increasing SA concentration enhances the proportion of stress granules positive cells in both cell lines until reaching the maximum at 100 μM for MDA-MB-231 (97.5 ± 0.6%) and 50 μM for SH-SY5Y (93.4 ± 4.2%).

Then to investigate the role of cholesterol and gangliosides, two major components of lipid rafts, in stress granules regulation we take advantage of methyl-β-cyclodextrin (MβCD) that removes cholesterol from the plasma membrane^33^ and D,L-threo-L-Phenyl-2-hexadecanoylamino-3-morpholino-1-propanol (PPMP) that interferes in the ganglioside synthesis pathway.^34^ Under both treatments, cells have reduced ability to assemble stress granules regardless cell lines (Figure 1(a) and (b)). PPMP and MβCD decrease similarly the cell ability to induce stress granules in SH-SY5Y cells (PPMP −20.8 ± 2.1% at 25 μM; MβCD −22.3 ± 5.5% at 25 μM and −15.9 ± 5.2% at 50 μM). No difference for either treatment under 100 μM sodium arsenite was observed. On MDA-MB-231 cells, PPMP treatment decreases the number of stress granules positive cells under 50 and 100 μM SA to 13.7 ± 2.3% and 22.6 ± 7.5% respectively. Whereas MβCD treatment decreases drastically the cell ability to assemble stress granules (−20.0 ± 5.3, 34.9 ± 18.3, and 49.5 ± 18.8% at, respectively, 50, 100, and 200 μM SA).

**Fig. 2:**
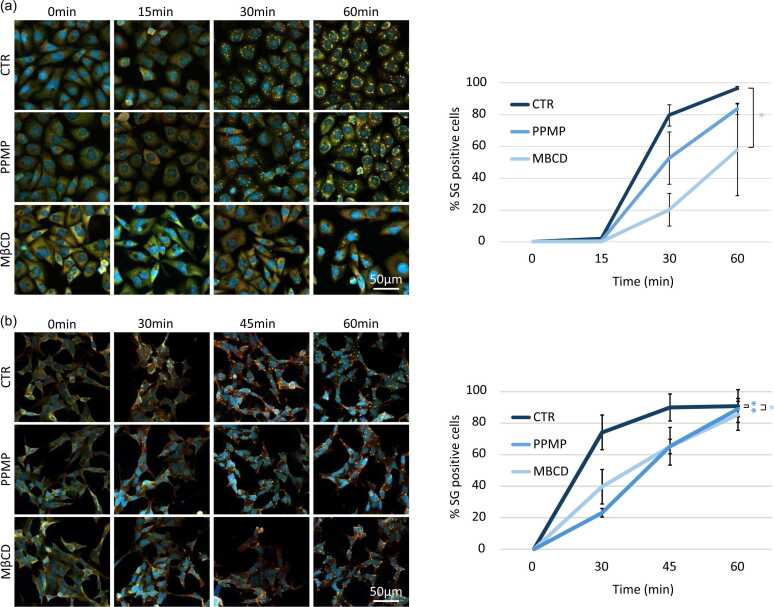
**Lack of gangliosides and cholesterol at the membrane delay the formation of stress granules.** (a) MDA-MB-231 cells are treated with PPMP (5 μM, 48 h) or MβCD (5 mM, 24 h) before stress granule experimentation. The day of the experimentation cells were treated with 200 μM sodium arsenite and collected at the indicated time. (b) SH-SY5Y cells are treated with PPMP (10 μM, 48 h) or MβCD (1 mM, 24 h) before stress granule experimentation. The day of the experimentation cells were treated with 100 μM sodium arsenite and collected at the indicated time. (a) and (b) After collection cells are then fixed and stained with G3BP1 (green), Caprin-1 (red) and DAPI (bleu) before to by imaged using confocal microscopy. Left: representative pictures. Right: quantifications. N ≤ 3, *P < 0.05. **P < 0.001. Abbreviation used: PPMP, D,L-threo-L-phenyl-2-hexadecanoylamino-3-morpholino-1-propanol.

The cells with altered cholesterol and gangliosides composition of lipid rafts are less likely to assemble stress granules. We also investigate if altered cholesterol and gangliosides composition was able to delay the assembly of stress granules at the highest sodium arsenite concentration. We quantify stress granules assembly after 15-, 30-, and 60-min exposure to 200 µM sodium arsenite for MDA-MB-231 cells. On basal condition, where cholesterol and gangliosides are not altered, stress granules assembly starts after 15 min of SA exposure (1.9 ± 0.4%) and rapidly increases over time (30 min, 79.6 ± 6.5%; 60 min 96.8 ± 0.8%) (Figure 2(a)). Stress granules assembly slows down when cholesterol and gangliosides levels within the membrane are reduced. When cholesterol is removed under MβCD treatment, no stress granules are observed after 15 min exposure to 200 µM SA and only 20.1 ± 10.4% and 57.8 ± 28.8% of cells assemble stress granules after respectively 30 and 60 min to SA 200 µM (Figure 2(a)). When the ganglioside biosynthesis pathway is disrupted, the stress granules assembly kinetic also slows down with 0.6 ± 0.6% of stress granules positive cells after 15 min exposure to 200 µM SA, 52.8 ± 16.5% after 30 min and 83.6 ± 3.6% after 60 min exposure to 200 µM SA (Figure 2(a)). For SH-SY5Y cells the assembly does not start before 30 min (data not shown) and follows the same trend as the MDA-MB-231 cells. PPMP treatment delays the assembly of stress granules of 45.7 ± 11.5% and 29.7 ± 12.1% and MβCD of 33.3 ± 7.4% and 29.9 ± 3.4% after an exposure of 30 and 45 min to 100 μM SA respectively (Figure 2(b)).

**Fig. 3:**
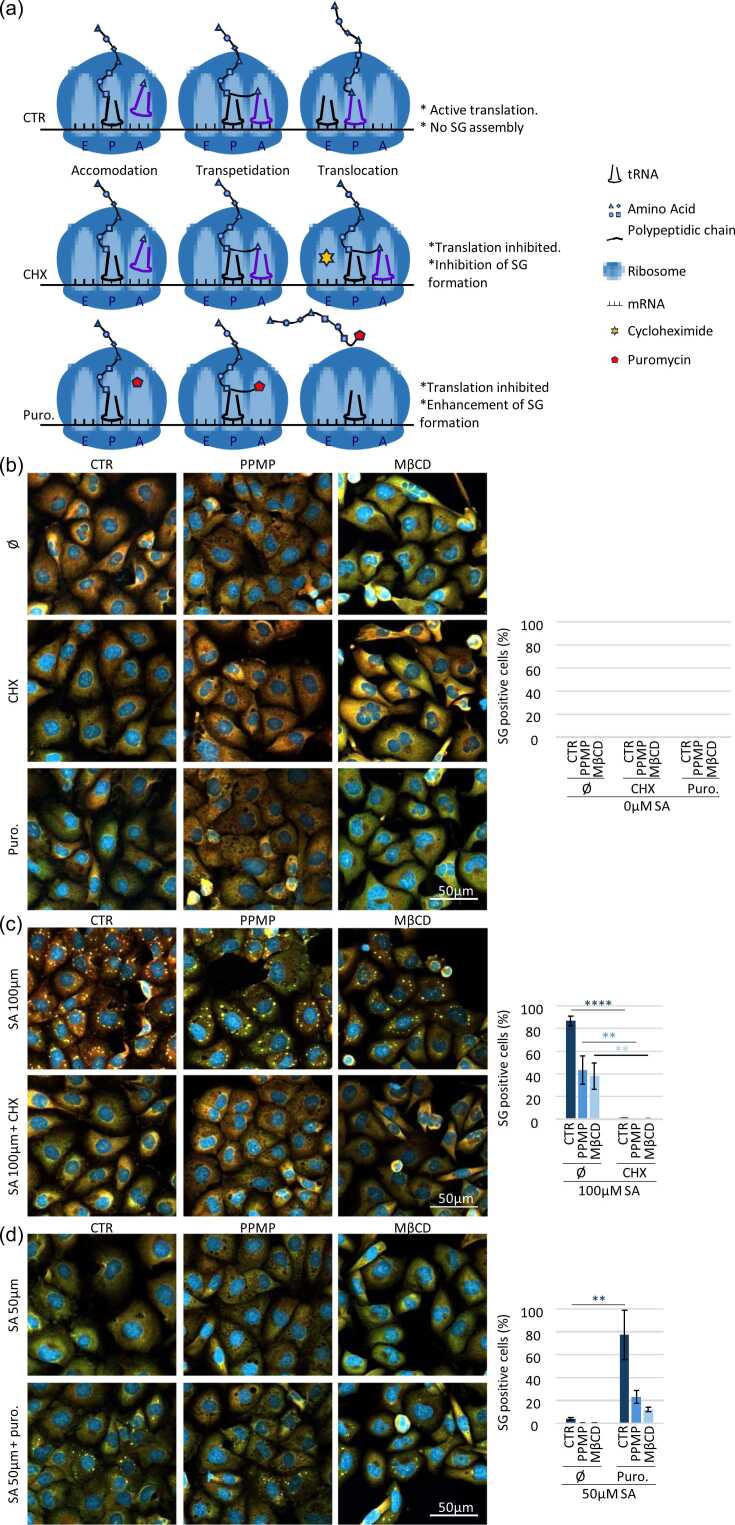
**Foci induced after lipid raft disruption are stress granules.** (a) Mechanism of action on translation of cycloheximide and puromycin. CTR) Translation in basal condition: 1) tRNA binds in the A binding site (acceptor). 2) Peptide bond then forms between the new amino acid and the forming peptide chain in the P (Polypeptide) site. 3) The tRNAs translocate to the E (Exit) site, where the first tRNA can exit the ribosome. CHX) Inhibition of translation by cycloheximide: When cycloheximide is present, it binds to the E site of the ribosome. As a result, the translocation step cannot take place, and translation stops with the RNAs being translated blocked in the ribosome. Puro.) Inhibition of translation by puromycin: When cells are treated with puromycin, the latter integrates with nascent polypeptide chains, stopping translation and releasing mRNAs and polypeptide chains into the cytoplasm. (b-d) MDA-MB-231 are treated with PPMP (5 μM, 48 h) or MβCD (5 mM, 24 h) before stress granule experimentation. Stress granule experimentation is the indicated combination of cycloheximide (CHX, 50 μM) or puromycin (20 μM) and/or SA (50 μM or 100 μM as indicated) for 1 h.Ø: no cycloheximide, no puromycin. Cells are then fixed and stained with G3BP1 (green), Caprin-1 (red) and DAPI (bleu) before to by imaged using confocal microscopy. Left: representative pictures. Right: quantifications. N = 3, **P < 0.01, ****P < 0.001. (b) Cells are treated with cycloheximide, puromycin and compared to untreated samples (Ø) without any SA addition. (c) All samples are stressed using 100 μM of SA to induce a robust stress granule response (Ø). Cycloheximide is added to inhibit stress granule formation. (d) Cells are treated with suboptimal stress (50 μM) to induce minimum or no stress granule (Ø). Puromycin is added to enhance stress granule formation. Abbreviations used: CHX, cycloheximide; Puro., puromycin; SA, sodium arsenite.

To ensure that foci observed under PPMP and MβCD are bona fide stress granules and not unspecific aggregation,^2^ we subject cells to puromycin that enhances stress granules formation by releasing mRNA into the cytoplasm^32^, ^35^ or cycloheximide that inhibits stress granules formation by trapping mRNA in the ribosome^32^, ^36^ (see Figure 3(a) for more detailed explanation on the mechanisms of these drugs). Without addition of SA, neither the puromycin nor the cycloheximide induce the stress granules assembly in any of our samples (Figure 3(b)). Control, PPMP or MβCD treated cells assemble stress granules under 100 μM SA, and this assembly is inhibited by the cycloheximide (Figure 3(c)). Meanwhile, stress granules formation is enhanced by the puromycin treatment in all cells treated with 50 μM SA (Figure 3(d)). The cell response to puromycin and cycloheximide treatment on top of the double labeling with G3BP1 and Caprin-1 refers to the formation of bona find stress granules in cells depleted in ganglioside or cholesterol at the membrane.

**Fig. 4:**
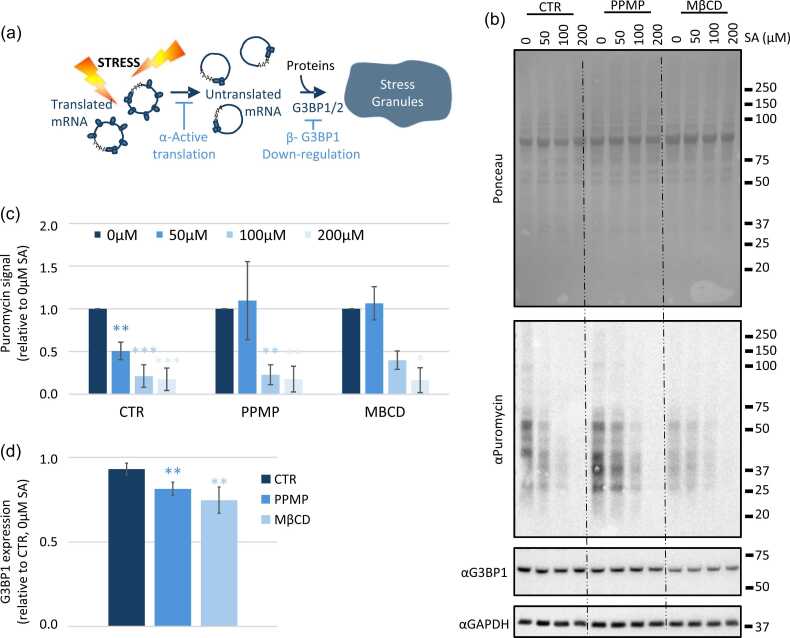
**Modification of lipids raft decrease G3BP1 expression level and translation inhibition in response to stress. (a) Under stress exposure, polysome disassemble to allow mRNA to be recruited to stress granule with proteins.***a-* Keeping the translation active will prevent the assembly of SG. *b-* The downregulation of one of the two scaffolding protein G3BP1 or G3BP2 will also (partially) prevent the formation of stress granule. (b-d) MDA-MB-231 cells are treated with PPMP (5 μM, 48 h) or MβCD (5 mM, 24 h) before to be exposed to the indicated sodium arsenite (SA) concentration for 1 h. 5 min before cell lysis, cells were pulsed with puromycin 5 μg/ml. For all samples, 15 μg of total protein are loaded for each sample. N ≤ 3, *P < 0.05, **P < 0.01, ***P < 0.005. (b) Representative blots. (c) and (d) Quantifications. Signal intensity was measured using ImageJ software. Each band intensity was expression relatively to the GAPDH intensity of the same sample and then expression was plotted relatively to the untreated sample (CTR, 0 μM SA). (c) Puromycin level, representative of general protein expression. (d) G3BP1 expression. All the CTR, PPMP and MBCD treated samples were, respectively, pulled together for analysis. Abbreviations used: MβCD, methyl-β-cyclodextrin; PPMP, D,L-threo-L-phenyl-2-hexadecanoylamino-3-morpholino-1-propanol.

Stress granules formation is a well-regulated process. Currently, two major pathways are known to regulate the formation of stress granules; by avoiding the translation repression^37^ or by affecting the level of expression of the level of expression of the stress granules core proteins G3BP1/2^5^, ^7^ (Figure 4(a) and (b)). We choose to investigate both options. First, we investigate the translation repression happening following stress exposure by using the puromycylation technic.^38^ Cells are pulse 5 min with low concentration of puromycin that will incorporate into nascent polypeptidic chains (Figure 3(b) and (c)). Puromycin detection using a specific antibody is representative of active translation. For the control sample the translation repression occurs already at 50 μM SA, whereas under PPMP and MβCD treatments a higher dose of SA (100 μM) is required to induce translation repression compared to the prestressed sample. On top, treatments with PPMP and MβCD reduce the G3BP1 global level of expression (Figure 3(b) and (d)).

## Discussion

Currently the stress granules field is still intensively investigating the link between stress granules and dysregulated biological process.^39^ Especially how some proteins mutations could affect the assembly and/or the function of stress granules in human pathogenesis. But recent studies show that lipids dysregulation is of growing interest in the development of human diseases.^20^, ^40^, ^41^, ^42^ This suggests that the role of lipids in regulatory pathways might have been underestimated and prompting us to investigate their implication in the regulation of stress granules assembly.

The present study, supported by complementary data in the recent literature, is part of the starting point of a new regulatory aspect of stress granules. Our data show that decreased synthesis of gangliosides inhibits the formation of stress granules. Whereas obesity, that is linked to an increased concentration of gangliosides,^43^ also enhances the probability to assemble stress granules in pancreatic cancer.^44^ Overall, this demonstrates that a fine modulation of gangliosides is necessary to have an efficient stress response and a healthy organism. On the other hand, we show that MβCD reduces the cell ability to induce stress granules. Although increasing cholesterol level in pituitary gland cells induces the formation of stress granule without further stress exposition,^24^ confirming a correlation between cholesterol level and cell ability to induce stress granules formation. Altogether we show that the deregulations of both gangliosides and cholesterol decrease the cell stress sensitivity to activate stress granules formation.

Under stress, those cells need a higher dose of oxidative stress to induce translation inhibition, the starting point for the stress granules assembly cascade.^11^, ^45^ Removing cholesterol and decreasing gangliosides levels in plasma membrane of cells result in a reduction of G3BP1 expression level, one of the key scaffolding proteins for stress granules assembly.^4^, ^5^ The combination of the two leads to a delay in the formation of stress granules and reduces cell sensitivity to stress.

Stress granules are known as prosurvival entities to help cell to overcome stress exposure/insult.^8^, ^9^, ^10^, ^16^, ^24^, ^46^ We then speculate that cells, *via* the dysregulation of their lipid composition within the plasma membrane, may become more vulnerable to stress exposure. Our data are particularly relevant in the context of neurodegenerative disorders where stress granules assembly is reduced, and lipid composition of neuronal cells is profoundly modified.^47^, ^48^ On the other hands the over-representation of cholesterol^22^, ^23^ together with change of gangliosides nature and level in cancer cells^20^ could increase the ability of cells to answer to stress exposure and provide prosurvival properties to those cells.

## Conclusions

This study highlights the fact that cholesterol and gangliosides dysregulations impact, directly or indirectly, the prosurvival mechanisms. This mechanism has been underestimated so far and raises several questions regarding its capacity to contribute to human pathogenesis *via* stress granules regulation as well as the precise underlying mechanisms. Similarly, since gangliosides and cholesterol are major component of membrane lipid raft.^49^, ^50^ With the current data, we cannot determinate if the effect we see on stress granules formation is dependent or independent of the structural rearrangement of the lipid rafts within the plasma membrane due to changes in cholesterol and ganglioside levels. We cannot rule out the global effect of lipid raft into stress granules formation. Further studies will be warranty on this aspect.

## Materials and methods

### Cell culture and cell treatment

MDA-MB-231 (ATCC) and SH-SY5Y (ATCC) cells are maintained at 37 °C with 5% CO_2_ in Gibco Dulbecco's Modified Eagle Medium: Nutrient Mixture F12 (DMEM-F12, GIBCO, Waltham, MA, USA) supplemented with 10% Fetal Bovine Serum (GIBCO, Waltham, MA, USA), 20 mM HEPES (GIBCO, Waltham, MA, USA), 1X Penicillin streptomycin (GIBCO, Waltham, MA, USA). Cells are treated with MβCD (MDA-MD-231 5 mM, SH-SY5Y 1 mM) 48 h before experimentation, or with PPMP (MDA-MD-231 5 μM, SH-SY5Y 10 μM) for 24 h.

### Immunofluorescence

Cells are seeded on coverslips, treated 48 h with PPMP or 24 h with MβCD before the experiment. After stress treatment, cells are washed quickly with PBS before to be fixed for 15 min with 4% Paraformaldehyde (Thermo Scientific, Waltham, MA, USA) in PBS. Cells are then permeabilized and blocked with IF buffer PBS-0.3% TX100 (Euromedex, Souffelweyersheim, France), 1% Glycine (Sigma, Saint-Louis, MO, USA), 5% Normal Horse Serum (Sigma, Saint-Louis, MO, USA), 5% Bovine Serum Albumin (Sigma, Saint-Louis, MO, USA) for 30 min at room temperature. Primary antibodies (Table S1) are diluted in IF buffer and incubated 1 h at room temperature. Coverslips are washed three times for 5 min with 1X PBS between primary and secondary antibody incubations. Subsequently, secondary antibodies (Table S1) are added along with DAPI for 1 h at room temperature in IF buffer. Cells were washed extensively 3 times with 1X PBS and mounted with ProLong Antifade reagent (Invitrogen, Carlsbad, CA, USA). Pictures are taken with confocal microscope LEICA LSM880.

### Western blot

Following drug(s) treatment(s), cells are washed with phosphate-buffered saline (PBS) and lysed in RIPA buffer (150 mM NaCl, 50 mM Tris pH7.4, 1%TritonX100, 0.1% SDS, 1% Sodium deoxycholate) with Halt phosphatase and protease inhibitors (Thermo Scientific). Laemmli's sample buffer supplemented is added to samples to 1X final concentration. For all experiments 15 μg of total protein are loaded on the gel. Samples are boiled, 5 min 95 °C before being loaded on a NuPAGE™ 4–12% Bis-Tris gel (Invitrogen) and transferred to nitrocellulose membrane (GE Healthcare). Membranes are blocked with Tris-buffered saline with 0.1% Tween-20 (TBS-T) with 5% BSA for at least 30 min at room temperature. Antibodies are diluted in 2.5% BSA in TBS-T. Primary antibodies are incubated overnight at 4 °C and secondary antibodies for 1 h at room temperature; mouse anti G3BP1 antibody (Santa Cruz sc-365338), rabbit anti Caprin-1 antibody (ProteinTech Group 15112-1-AP), mouse anti puromycin antibody (Millipore MABE342), mouse anti GAPDH (Abcam ab8245). Antibody detection is performed using SuperSignal West Pico Chemiluminescent Substrate (Thermo Scientific). Revelation of the blot is made using G:BOX machine (Syngene) *via* the GeneSys software. Blot analysis and quantification are done using ImageJ software.

### Statistical analysis

Statistical analyses are done on 3 independent experiments. One way or two ways analysis of variance were performed as mentioned in the figure legend: *P < 0.05, **P < 0.01, ****P < 0.0001.

## CRediT authorship contribution statement

**Coralie Di Scala:** Writing – Review & editing, Writing – Original draft, Resources, Funding acquisition, Conceptualization. **Anaïs Aulas:** Writing – Review & editing, Writing – Original draft, Validation, Project administration, Methodology, Formal analysis, Conceptualization.

## Declarations of interest

The authors declare that they have no known competing financial interests or personal relationships that could have appeared to influence the work reported in this paper.

## Data Availability

No data were used for the research described in the article.

## Footnotes

Appendix A Supplementary data associated with this article can be found online at doi:10.1016/j.cstres.2025.100093.
